# A Call for Randomized Controlled Trials to Test the Efficacy of Chloroquine and Hydroxychloroquine as Therapeutics against Novel Coronavirus Disease (COVID-19)

**DOI:** 10.4269/ajtmh.20-0230

**Published:** 2020-04-03

**Authors:** Maryam Keshtkar-Jahromi, Sina Bavari

**Affiliations:** 1 Division of Infectious Diseases, Johns Hopkins University School of Medicine, Baltimore, Maryland;; 2 Edge BioInnovation Consulting, Frederick, Maryland

Novel coronavirus disease (COVID-19) is spreading fast around the world, with many uncertainties about treatment and prevention. Currently, there are no U.S. Food and Drug Administration (FDA)–approved drugs for the treatment of patients with COVID-19. A great deal of effort is ongoing to find effective therapeutics and preventive measures against this transmissible virus with high mortality. Available data are limited, and there are minimal randomized controlled trial (RCT) data on the efficacy of antiviral or immunomodulatory agents for the treatment of COVID-19.

Chloroquine (CQ) and hydroxychloroquine (HCQ) have been used to treat malaria for 70 years. Recently, triggered in part by media reports on potential efficacy, CQ and HCQ have been widely used off-label for treatment and prevention of COVID-19. These drugs were suggested for clinical usage after in vitro activity was observed against COVID-19.^1,2^ The molecular mechanism is believed to involve action at multiple steps in the viral pathway, including cellular entry and exit. These drugs alter intracellular pH, and may induce endoplasmic reticulum stress, causing misformation of essential viral proteins. However, in vitro activity of these drugs should not be interpreted as proof of clinical efficacy against COVID-19. Similar in vitro activity of CQ and HCQ was identified against multiple other viruses, but follow-up clinical trials did not show significant clinical efficacy of these drugs, for example, against Ebola virus disease,^3^ chikungunya,^4^ influenza,^5^ HIV infection,^6^ and dengue.^7^

A report of a nonrandomized trial of 20 COVID-19 patients in France who received HCQ alone or in combination with azithromycin showed that, compared with untreated controls, HCQ reduced nasopharyngeal viral carriage 6 days after the initiation of therapy.^8^ Another preliminary report containing limited information noted that, in 100 COVID-19 patients in China, CQ offered superior clinical efficacy than controls.^9^ Based on these limited data, the National Health Commission of the People’s Republic of China is considering CQ in their national guidelines to treat COVID-19.^9^ If efficacy of CQ and HCQ are demonstrated by RCTs, this would be the first time they are found to be effective for the treatment of a viral infection.

An analysis of the clinical trials conducted during the 2014–2015 Ebola outbreak in West Africa showed that an RCT was an ethical, appropriate, and efficient path toward identification of safe and efficacious therapeutics.^10^ The equipoise for a placebo-controlled RCT for the treatment of COVID-19 derives from the absence of proven therapy for COVID-19 and the need to establish benefit versus harm caused by any experimental therapeutic. This ethical consideration justifies initiation of clinical trials; many are planned or are underway to study CQ and HCQ for treating or preventing COVID-19 in different countries (as examples: NCT04315896,^11^ NCT04318015,^12^ NCT04318444,^13^ NCT04321278,^14^ NCT04308668,^15^ NCT04304053,^16^ NCT04316377,^17^ and NCT04303299^18^). In the meantime, significant off-label use is occurring globally, including in many U.S. hospitals, with not only potential benefit but also potential risk of harm, whereas adequate data on efficacy and safety are not yet available.

Many academic institutions in the United States and overseas have drafted institutional guidelines for off-label use of drugs for COVID-19, including CQ and HCQ with different dosages and duration for either treatment or prophylaxis, but there is no standard recommendation for prescribing these medications for this disease. Moreover, CQ and HCQ may cause harm, with narrow therapeutic windows, and many side effects, including cardiac toxicity (QT prolongation, torsade de pointes, and ventricular arrhythmia), which may be particularly problematic in the elderly, who are also most likely to suffer from severe COVID-19.^19^ Coronavirus disease also appears to cause cardiac effects, including myocarditis.^20^ Other side effects associated with CQ and/or HCQ include retinopathy, nausea, vomiting, bone marrow suppression, psychosis, seizure, emotional lability, vertigo, dizziness, and myopathy.^21^ The known toxicities of CQ and HCQ raise concern regarding toxicity of self-administered drugs, with overdoses, severe toxicity, and death described recently in the popular press.

Although the COVID-19 pandemic is global, it may be a particular burden for developing countries with limited infrastructure. Given a lack of evidence and intense pressure to try something in COVID-19 patients, clinicians may increasingly turn to off-label usage of drugs. We call on public health organizations to urgently consider creating or expanding partnerships with local governments to support unified RCTs to test the efficacies of potential therapeutics against COVID-19. Leveraging the previously developed adaptive RCT design from the National Institute of Allergy and Infectious Diseases (NIAID) PALM trial, which efficiently assesses multiple arms against a common comparator, the NIAID, National Institute of Health, and the WHO have already launched COVID-19 RCTs. The NIAID trial is a multinational placebo-controlled trail of 440 hospitalized COVID-19 patients, initially comparing remdesivir with placebo.^22^ The WHO solidarity trial is a large, adaptive, five-arm trial comparing four promising COVID-19 regimens: remdesevir, CQ, lopinavir–ritonavir, and lopinavir–ritonavir plus interferon beta, all compared with standard of care, with mortality as the primary end point.^23^ Both trials require close data and safety monitoring board oversight and allow for dropping poorly performing arms and adding promising putative therapeutics as they are developed. So far, many countries, including Argentina, Bahrain, Canada, France, Iran, Norway, South Africa, Spain, Switzerland, and Thailand have signed up for the trial. Both trials are examples of robust efforts to generate high-quality evidence to identify medicines that may potentially save lives in the global battle against COVID-19.

The off-label use of CQ and HCQ to treat or prevent COVID-19 must be cautious, considering potential serious toxicities. Global multicenter RCTs testing safety and efficacy of CQ or HCQ seem to be the most reasonable plan to urgently gather data on the efficacy and safety of these medications in the treatment of COVID-19. Before the availability of robust data from RCTs, we highly recommend that off-label use of medications to treat COVID-19, including CQ or HCQ, be accompanied by careful observation for potential toxicity.
